# Systematic Inflammatory Response Syndrome (SIRS)-Like Reaction Following the Infusion of the Unregulated IV Supplement Hemobex

**DOI:** 10.7759/cureus.98171

**Published:** 2025-11-30

**Authors:** Jace A Leslie, Michael Silberman, Vasilios Katsaitis

**Affiliations:** 1 Emergency Medicine, Ascension St. Thomas Rutherford/University of Tennessee Health Science Center Graduate Medical Program, Murfreesboro, USA; 2 Emergency Department, University of Tennessee Health Science Center, Murfreesboro, USA

**Keywords:** hemobex, sepsis, sirs, supplements, toxicology

## Abstract

Unregulated supplements are increasingly used for perceived health and fitness benefits, including parenteral formulations administered without medical oversight. Infusions of such products can trigger systemic inflammatory response syndrome (SIRS)-like reactions that mimic sepsis and may prompt full sepsis protocol activation. We report the case of a previously healthy 21-year-old male who, within 24 hours of receiving an IV “Hemobex” preparation (a vitamin B complex with liver extract) obtained outside of medical oversight, developed acute fever, tachycardia, gastrointestinal symptoms, encephalopathy, leukopenia, followed by marked leukocytosis, elevated transaminases, and lactic acidosis. Comprehensive infectious and toxicologic workup - including blood cultures, chest radiography, abdominal/pelvic CT, lumbar puncture, and viral studies - was unrevealing. He was managed with guideline-based supportive care and empiric broad-spectrum antibiotics. Both his clinical condition and laboratory abnormalities normalized rapidly by hospital day three.

The presentation is consistent with a cytokine-mediated SIRS-like reaction or culture-negative sepsis potentially triggered by an unregulated, animal-derivative-containing infusion. Regulatory authorities in Guatemala have issued warnings about “Hemobex” due to its lack of approval and reports of counterfeit products. Potential mechanisms include endotoxin contamination, cytokine release, and, less commonly, acute type I or type III hypersensitivity. Clinicians should maintain a high index of suspicion for toxic/immunogenic mimics of sepsis after unregulated IV supplement exposures, while continuing to manage patients according to sepsis guidelines until infection is ruled out. This report underscores the need for clinical vigilance and regulatory oversight in these scenarios.

## Introduction

Systemic inflammatory response syndrome (SIRS) often presents along a spectrum and is defined by the presence of at least two of the following four criteria: fever (temperature >38°C or <36°C), tachycardia (heart rate >90 bpm), tachypnea (respiratory rate >20 or PaCO₂ <32 mmHg), and abnormal white blood cell counts (leukocytosis >12,000/mm³ or leukopenia <4,000/mm³). If not promptly addressed, SIRS can progress to end-organ dysfunction and shock [1,2]. It is important to note that SIRS can have many etiologies and sources that are not clinically apparent. Moreover, its initial presentation often closely resembles other conditions, including infusion-related reactions and alternative causes of shock.

Although infusion reactions are generally well documented, the case described in this report presents multiple confounding factors that could implicate multiple pathophysiological pathways. One of the more commonly documented pathways is the anaphylactic type 1 hypersensitivity (T1H) pathway [3], which is clinically significant given its potential to precipitate cardiac arrest, respiratory compromise, and other severe consequences if not promptly recognized and managed [4]. Quick recognition of SIRS or other cytokine-mediated clinical presentations is critical to ensuring optimal clinical outcomes [5].

## Case presentation

A 21-year-old previously healthy male presented to the emergency department with an acute onset of fever, nausea, vomiting, diarrhea, diffuse abdominal cramping, and body aches. He denied chest pain, shortness of breath, or any past medical or surgical history. Symptoms developed within 24 hours after receiving an intravenous infusion of a supplement called “Hemobex” (Liver Extract + Vitamin B12) from a non-medical acquaintance, marking his first exposure to this product. He reportedly had taken this for supposed health benefits and recovery; however, our investigation revealed that there is very limited scientific data or credible information on this supplement, with references seemingly confined to a few online advertisements. [6].

Initial vital signs and physical exam findings were as follows: temperature: 101.4 °F (38.6 °C), heart rate: 115 bpm, respiratory rate: 22/min, blood pressure: 116/74 mmHg, and SpO_2_: 98% on room air. He was warm, diaphoretic, markedly uncomfortable, and fully oriented. Physical exam was otherwise nonfocal: no meningismus or rash; lungs clear; tachycardic but regular rhythm without murmurs. According to the Centers for Medicare & Medicaid Services SEP-1 criteria, the patient met sepsis criteria at triage [2]. The diagnostic workup included a complete blood count (CBC), comprehensive metabolic panel (CMP), creatine kinase (CK), serum lactate, blood cultures, respiratory viral panel (including influenza and SARS-CoV-2), chest radiograph, contrast-enhanced CT of the abdomen and pelvis, urine drug screen, and lumbar puncture with cerebrospinal fluid analysis (cell count, protein, glucose, bacterial culture, and viral/encephalitis panel).

**Figure 1:**
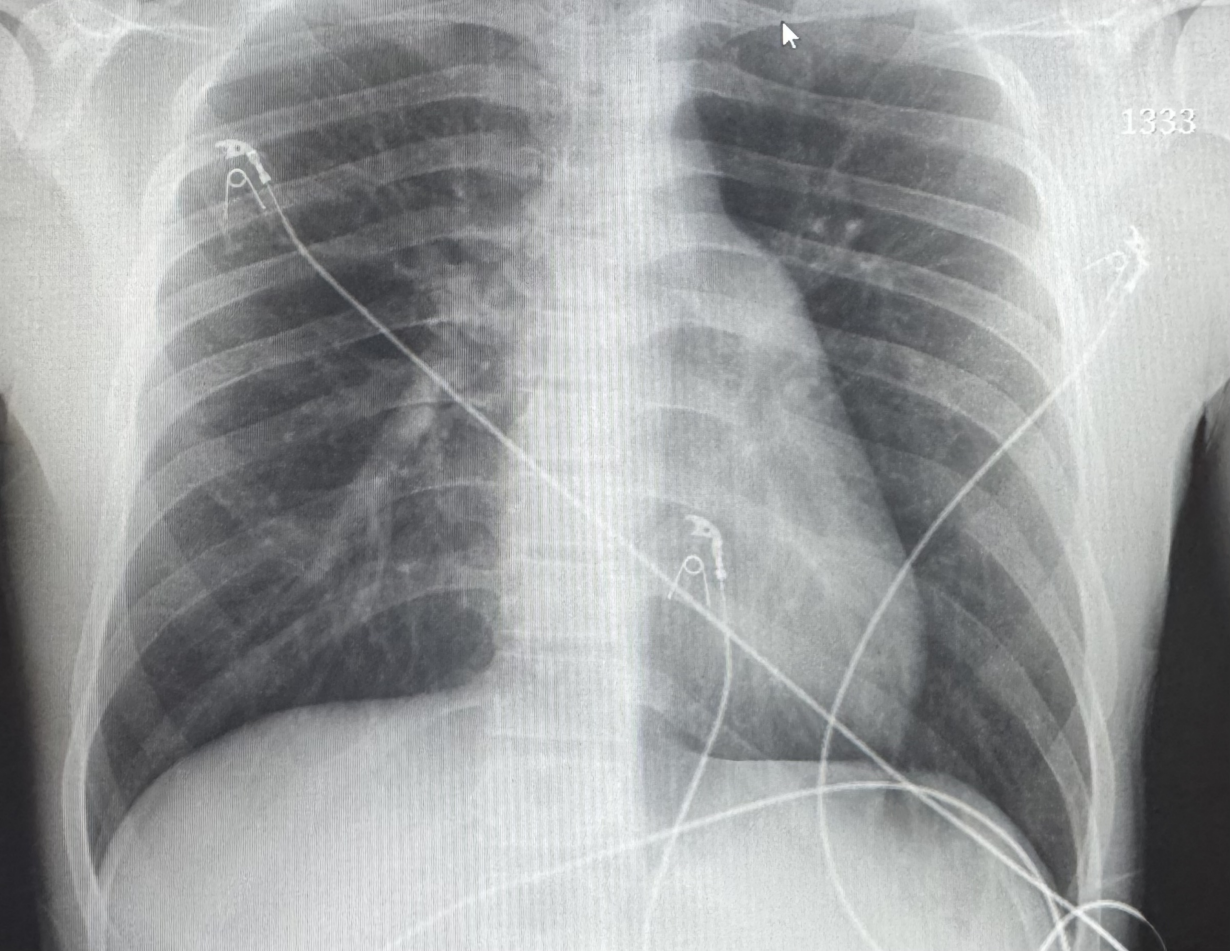
Chest X-ray from emergency department

**Figure 2:**
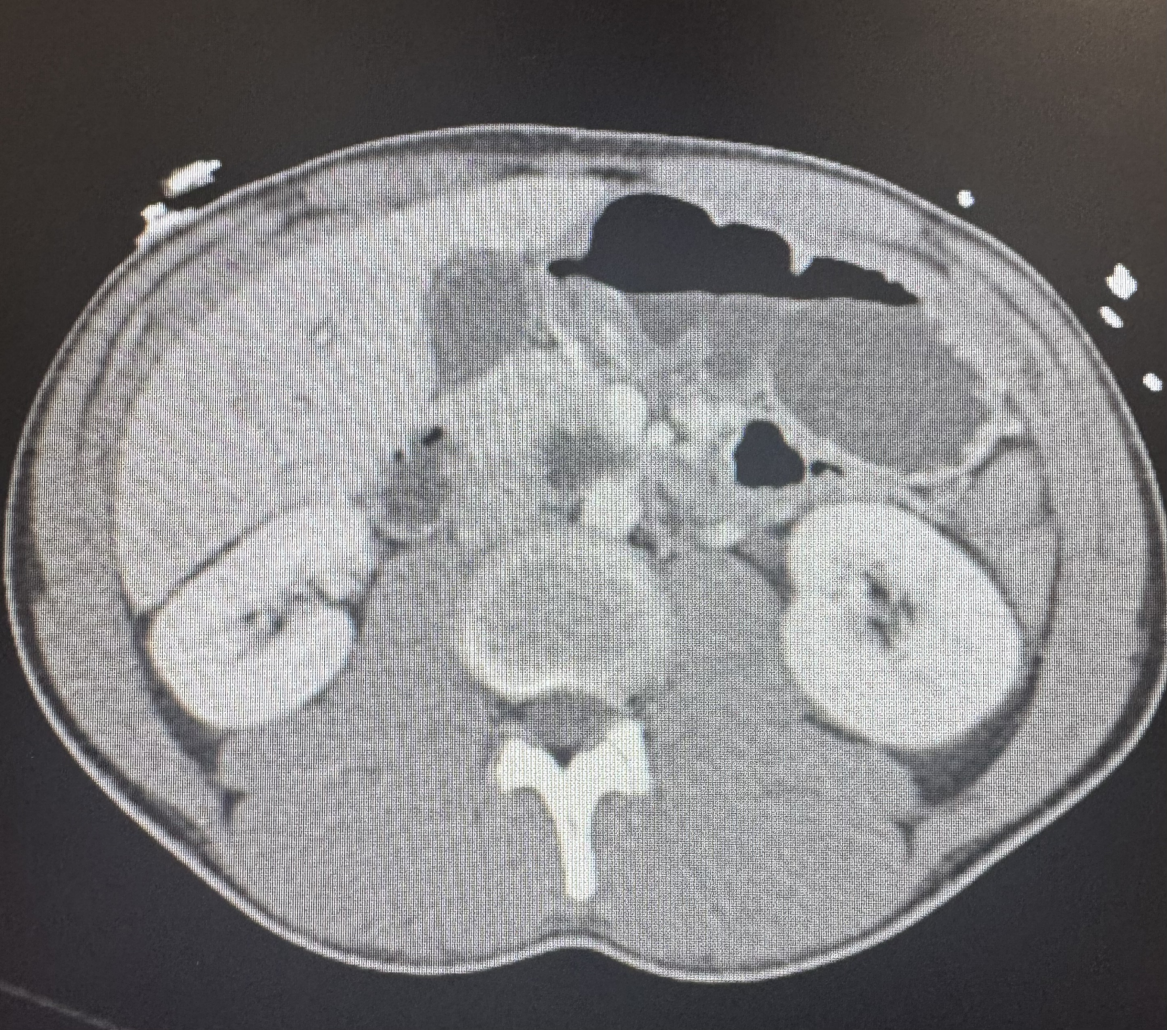
CT abdomen/pelvis with IV contrast CT scan of the patient's abdomen showing no evidence of intra-abdominal infection or inflammation CT: computed tomography

ED management included 1 L lactated Ringer’s, acetaminophen 1,000 mg, ondansetron 4 mg, vancomycin 1,250 mg IV, and ceftriaxone 2 g IV. Initial lab results were as follows: WBC: 2.0 × 10^3^/µL with 81% neutrophils; potassium: 3.3 mmol/L; aspartate aminotransferase (AST): 52 U/L; alanine aminotransferase (ALT): 22 U/L; lactate: 2.3 mmol/L; and CK: 206 U/L. Chest radiograph (Figure 1) and abdominal CT (Figure 2) showed no acute pathology. The patient’s symptoms improved following administration of antipyretics and IV fluids, and he was admitted for observation and further evaluation.

Hospital course

Two hours after the initial ED assessment, the patient’s lactate levels increased to 3.1 mmol/L from 2.3 mmol/L. Three hours after the initial CBC, the WBC increased to 27.3 × 10³/µL, and lactic acid rose to 6.0 mmol/L. On hospital day two, laboratory values showed potassium 3.4 mmol/L, AST 223 U/L, ALT 111 U/L, and INR 1.8. Blood cultures, cerebrospinal fluid studies (cell counts, culture, and PCR-based panels), and respiratory/encephalitis viral panels remained negative. The patient developed transient encephalopathy and herpes labialis, for which acyclovir was initiated, along with a liter of IV fluids. He continued receiving the antibiotics started in the emergency department, including vancomycin and ceftriaxone.

On hospital day three, the patient’s symptoms had resolved, vital signs normalized, and laboratory values improved, including WBC 15.9 × 10³/µL, potassium 4.0 mmol/L, AST 34 U/L, ALT 56 U/L, and lactate 1.1 mmol/L. Antibiotics were discontinued given the absence of bacterial infection and negative blood cultures by day three. He was discharged with supportive care instructions and counseling to avoid unregulated supplements, which he acknowledged and understood. The patient has not returned for follow-up, and no additional records regarding his presentation are available at this time. A timeline of his treatment course is summarized in Table 1.

**Table 1:** Lab values Patient lab values are presented from right to left, showing results from day one in the emergency department, followed by day one through day three of the hospital admission, up until the patient’s discharge BUN: blood urea nitrogen; GFR: glomerular filtration rate; AST: aspartate aminotransferase; ALT: alanine transaminase; THC: delta-9-tetrahydrocannabinol; UA: urinalysis; WBC: white blood cell; RBC: red blood cell; MCV: mean corpuscular volume; MCH: mean corpuscular hemoglobin; MCHC: mean corpuscular hemoglobin concentration; HSV: herpes simplex virus; CSF: cerebrospinal fluid

Variable	Hospital day 3	Hospital day 2	Hospital day 1		Emergency room day 1
Sodium (normal 136-145 mmol/L)	137 mmol/L	140 mmol/L	136 mmol/L		139 mmol/L
Potassium (normal 3.5-5.1 mmol/L)	4.0 mmol/L	3.4 mmol/L L	3.4 mmol/L L		3.3 mmol/L L
Chloride (normal 98-107 mmol/L)	106 mmol/L	128 mmol/L H	106 mmol/L		104 mmol/L
Co_2_ (normal 22-29 mmol/L)	22 mmol/L	17 mmol/L L	21 mmol/L L		20 mmol/L L
BUN (normal 7-26 mg/dL)	11 mg/dL	7 mg/dL	14 mg/dL		15 mg/dL
Creatinine (normal 0.7-1.3 mg/dL)	0.8 mg/dL	0.6 mg/dL L	1.0 mg/dL		1.0 mg/dL
Estimated GFR (normal ≥60 mL/min/1.73 m^2^)	129 mL/min/1.73 m^2^	141 mL/min/1.73 m^2^	104 mL/min/1.73 m^2^		105 mL/min/1.73 m^2^
Creatinine clearance	128 mL/min	171 mL/min	97 mL/min		108 mL/min
Glucose (normal 70-105 mg/dL)	93 mg/dL	79 mg/dL	148 mg/dL H		97 mg/dL
Calcium, serum (normal 8.4-10.2 mg/dL)	8.6 mg/dL	5.3 mg/dL C	7.6 mg/dL L		8.8 mg/dL
Total protein (normal 6.0-8.3 gm/dL)	7.4 gm/dL	4.0 gm/dL L	6.0 gm/dL		8.2 gm/dL
Albumin (normal 3.5-5.2 gm/dL)	3.7 gm/dL	2.2 gm/dL L	3.4 gm/dL L		4.6 gm/dL
Bilirubin (normal 0.2-1.2 mg/dL)	0.3 mg/dL	0.3 mg/dL	0.9 mg/dL		1.0 mg/dL
Alkaline phosphatase (normal 40-150 U/L)	89.0 U/L	49.0 U/L	74.0 U/L		88.0 U/L
AST (normal 5-34 U/L)	34 U/L	35 U/L H	223 U/L H		52 U/L H
ALT (normal Low- <=55 U/L)	56 U/L H	46 U/L	111 U/L H		22 U/L
Anion gap (normal 6-17 mmol/L)	9 mmol/L	-5 mmol/L L	9 mmol/L		15 mmol/L
Lactic acid (normal 0.5-2.2 mmol/L)	1.1 mmol/L	2.6 mmol/L H	3.3 mmol/L H	6.0 mmol/L C	2.3 mmol/L H
Vitamin B12 (normal 213-816 pg/mL)		764.0 pg/mL			
Creatinine kinase (normal 30-200 U/L)		75 U/L			203 U/L
HIV I and II		Non-reactive			
Hepatitis A IgM		Non-reactive			
Hepatitis B core IgM		Non-reactive			
Hepatitis Bs Ag		Non-reactive			
Hepatitis C Ab		Non-reactive			
Amphetamine/methamphetamine					Negative
Barbiturates					Negative
Benzodiazepines					Negative
Cocaine w/metab					Negative
Opiates					Negative
Phencyclidine					Negative
THC					Negative
UA Color					Yellow
UA appear					Clear
UA specific gravity (normal 1.005-1.030)					1.006
UA pH (normal 5-7)					8
UA protein					Negative
UA glucose					Negative
UA ketones					Negative
UA bilirubin					Negative
UA blood					Negative
UA nitrite					Negative
UA urobilinogen (normal 0.2-1.0 Eu/dl)					0.2 Eu/dl
UA leukocyte esterase					Negative
WBC (normal 4.8-10.8 x 10^3^/mm^3^)	15.9 x 10^3/mm3 H	26.5 x 10^3^/mm^3 ^H	27.3 x 10^3^/mm^3 ^H		2.0 x 10^3^/mm^3 ^L
RBC (normal 4.2-5.4 x 10^6^/mm^3^)	4.98 x 10^6^/mm^3^	4.08 x 10^6^/mm^3 ^L	4.42 x 10^6^/mm^3^		5.48 x 10^6^/mm^3 ^H
Hemoglobin (normal 14-18 gm/dL)	14.2 gm/dL	11.9 gm/dL L	12.8 gm/dL L		16.0 gm/dL
Hematocrit (normal 40-52%)	43.30%	36.5 % L	38.6 % L		47.80%
MCV (normal 78-98 fl)	86.9 fl	89.5 fl	87.3 fl		87.2 fl
MCH (normal 26-34 pg)	28.5 pg	29.2 pg	29.0 pg		29.2 pg
MCHC (normal 32-36 gm/dL)	32.8 gm/dL	32.6 gm/dL	33.2 gm/dL		33.5 gm/dL
Red cell distribution (normal 11.5-14.5%)	12.50%	13.10%	12.90%		12.10%
Platelets (normal 130-400 x10^3^/mm^3^)	188 x 10^3^/mm^3^	141 x10^3^/mm^3^	142 x 10^3^/mm^3^		203 x 10^3^/mm^3^
HSV-1 DNA			Negative		
HSV-2 DNA			Negative		
Influenza A					Negative
Influenza B					Negative
SARS-CoV-2 source					Nasopharyngeal
SARS-CoV-2					Negative
Blood culture	Final report				
	No growth at 5 days				
	Preliminary report				
	No growth at 4 days.				
CSF culture	Final report				
	No growth at 3 days				
	Preliminary report				
	No growth at 2 days				
	Gram stain report				
	No white blood cells				
	No organisms seen				
	Reviewed by a microbiology tech				
Color CSF					Colorless
Clarity CSF					Clear
WBC CSF (normal 0-8 /CU MM)					0/CU MM
RBC CSF (normal 0-10 /CU MM)					1/CU MM
CSF glucose (normal 40-70 mg/dL)					66 mg/dL
CSF protein (normal 15-45 mg/dL)					21.3 mg/dL

The patient’s family provided smartphone photos of the infused product packaging (see Appendices), which appeared consistent with “Hemobex,” a vitamin B complex containing added liver extract that is not approved for intravenous administration.

## Discussion

This report illustrates a sepsis-like presentation following exposure to an unregulated parenteral supplement. The initial leukopenia with subsequent rebound leukocytosis, rising then normalizing lactate, transient coagulopathy (INR 1.8), and reversible transaminitis suggest a cytokine-driven SIRS-like syndrome or other inflammatory reaction. Public health alerts in Guatemala (October 2024) cautioned against the use of 'Hemobex' due to a lack of regulatory authorization, safety assurances, and quality controls, with reports of counterfeit products in circulation [7,8]. Although the patient’s specific vial could not be verified, the potential risks associated with non-authorized or counterfeit parenteral products remain significant.

Unregulated liver extract-containing formulations may harbor endotoxins or antigenic proteins provoking cytokine cascades, notably IL-6, IL-1β, and TNF-α, leading to vasodilation, capillary leak, hepatic dysfunction, and hematologic changes [9,10]. The transient transaminase elevations that resolved within 72 hours align with cytokine-mediated hepatocellular injury. The biphasic WBC pattern (early leukopenia, then leukocytosis) is consistent with early apoptosis/demargination followed by marrow mobilization seen in sepsis and cytokine release syndrome (CRS).

Another consideration is type III hypersensitivity, a reaction mediated by immune complex deposition after antigen exposure, as observed in serum sickness. Animal-derived extracts, such as those in Hemobex, may be antigenic, facilitating the formation of circulating immune complexes that deposit in tissues and activate complement. While this mechanism typically unfolds over a longer timeline and is less commonly associated with acute presentations, it may also contribute to the observed SIRS-like syndrome [11]. 

Culture-negative sepsis accounts for up to 30-50% of all sepsis cases, and blood cultures may be falsely negative due to intermittent bacteremia, prior antimicrobial exposure, or fastidious organisms [12]. Given the patient’s presentation and clinical course with fever, hypotension risk, encephalopathy, and lactic acidosis, early administration of broad-spectrum antibiotics was warranted in accordance with Surviving Sepsis Campaign guidelines [13]. While specific immunomodulatory therapies are used in oncology-related CRS, such interventions are not routine [14].

## Conclusions

An infectious source was never found, but the principle of "treating as sepsis until proven otherwise" was applied in this case. The management of CRS and other non-infectious SIRS-like conditions remains primarily supportive, with discontinuation and avoidance of the inciting agent being critical. IV fluid resuscitation, antipyretics, and monitoring in the inpatient setting are warranted until symptoms and labs resolve. Ultimately, the patient’s rapid improvement with supportive care, without identification of an infectious source, highlights the importance of recognizing toxic and immunogenic mimics of sepsis. This is especially important in the context of unregulated supplement use, which is becoming increasingly common. Emergency physicians should maintain a high index of suspicion for CRS and inflammatory syndromes in young, otherwise healthy patients presenting with sepsis-like physiology after exposure to over-the-counter or other unknown substances, while continuing to manage them according to established sepsis guidelines.

## Figures and Tables

**Figure 3:**
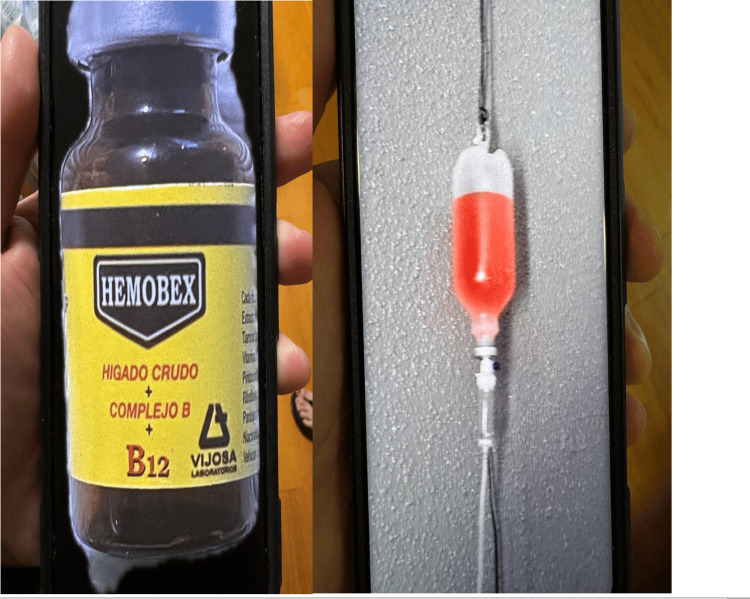
Photo of the IV supplement Photo of the IV supplement labeled "Hemobex" provided by the patient's family and the actual IV given to the patient

**Figure 4:**
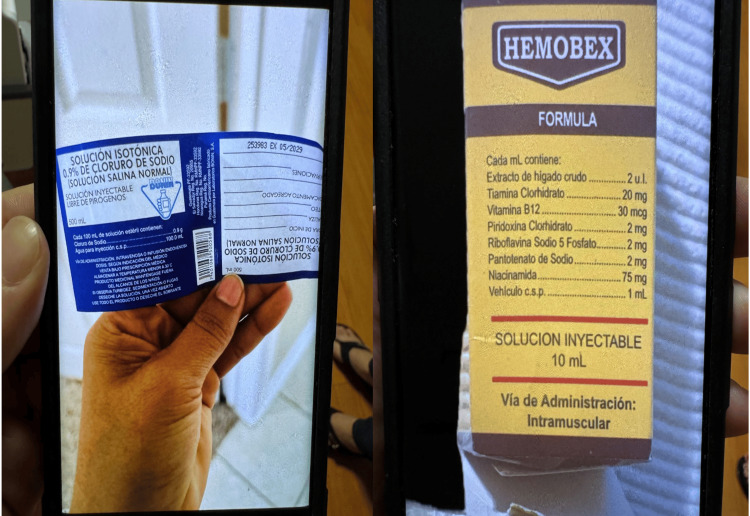
Photo of the label of the IV supplement Photo of the label of the IV supplement provided by the patient's family

## References

[1] Evans L, Rhodes A, Alhazzani W (2021). Surviving Sepsis Campaign: International guidelines for management of sepsis and septic shock 2021. Crit Care Med.

[2] Bone RC, Balk RA, Cerra FB (1992). Definitions for sepsis and organ failure and guidelines for the use of innovative therapies in sepsis. The ACCP/SCCM Consensus Conference Committee. American College of Chest Physicians/Society of Critical Care Medicine. Chest.

[3] Chitwood WR, Moore CD (1952). Anaphylactic shock following intravenous administration of vitamin B complex. J Am Med Assoc.

[4] Juel J, Pareek M, Langfrits CS, Jensen SE (2013). Anaphylactic shock and cardiac arrest caused by thiamine infusion. BMJ Case Rep.

[5] Zaikov S, Gumeniuk G, Veselovsky L (2021). The problem of hypersensitivity to vitamin preparations. Infus Chemother.

[6] (2025). HEMOBEX Liver Extract + Vitamin B12 10ml Vial 3-Pack (Injections). https://vitaminasdelsalvador.com/product/hemobex-vial-10ml-3pack-inyecciones-vitamina-b12/.

[7] Diario El Mundo (2025). El Mundo newspaper: warning about counterfeit “Hemobex” and how to identify it (Site in Spanish). https://diario.elmundo.sv/nacionales/alertan-sobre-falsificacion-de-medicamento-hemobex-y-asi-puede-identificarlo.

[8] (2025). Soy502. Liver medication “Hemobex” banned in Guatemala (Site in Spanish). https://www.soy502.com/articulo/medicamento-higado-prohibido-guatemala-100931.

[9] Shimabukuro-Vornhagen A, Gödel P, Subklewe M (2018). Cytokine release syndrome. J Immunother Cancer.

[10] Barbier L, Ferhat M, Salamé E (2019). Interleukin-1 family cytokines: keystones in liver inflammatory diseases. Front Immunol.

[11] Usman N, Annamaraju P (2023). Type III Hypersensitivity Reaction. Updated 22 May.

[12] Phua J, Ngerng W, See K (2013). Characteristics and outcomes of culture-negative versus culture-positive severe sepsis. Crit Care.

[13] Evans L, Rhodes A, Alhazzani W (2021). Surviving Sepsis Campaign: international guidelines for management of sepsis and septic shock 2021. Intensive Care Med.

[14] Shah D, Soper B, Shopland L (2023). Cytokine release syndrome and cancer immunotherapies - historical challenges and promising futures. Front Immunol.

